# Total arch replacement for an aortic arch aneurysm with cold agglutinin disease after rituximab and plasmapheresis

**DOI:** 10.1186/s13019-023-02388-x

**Published:** 2023-10-10

**Authors:** Atsuyuki Mitsuishi, Yujiro Miura, Kyosuke Saeki, Yoshinori Nomura, Katsumata Yoshifumi, Keisuke Yoshida

**Affiliations:** 1 Department of Cardiovascular Surgery Kochi Medical School, 185-1, Kohasu, Nankoku-shi, Okohcho, Kochi Prefecture 783-8505 Japan; 2 Department of Hematology Ehime Prefectural Central Hospital, Matsuyama-shi, Ehime Prefecture, Kasugamachi 790-0024, 83 Japan; 3 Department of Clinical Engineering Kochi Medical School, 185-1, Kohasu, Nankoku-shi, Okohcho, Kochi Prefecture 783-8505 Japan; 4 Department of Anesthesiology and intensive Care Medicine Kochi Medical School, 185-1, Kohasu, Nankoku-shi, Okohcho, Kochi Prefecture 783-8505 Japan

**Keywords:** Cold agglutinin disease, Rituximab, Plasmapheresis, Mild hypothermia, Total arch replacement, Thoracic aortic aneurysm, Frozen elephant trunk, Recirculation, Warm cardioplegia, Aortic dissection

## Abstract

**Background:**

Cold agglutinin disease can lead to significant complications, especially for patients undergoing arch repair requiring hypothermic circulatory arrest. Rituximab and plasmapheresis are treatments for cold agglutinin disease. However, its use in patients with Stanford type A dissection has not been reported. Therefore, after consultation with hematologists, we used rituximab and plasmapheresis before mild hypothermic aortic arch surgery to maintain the body temperature above the thermal altitude.

**Case presentation:**

This report describes an 86-year-old male patient with acute type A aortic dissection who received outpatient treatment for rheumatoid arthritis and a 55-mm thoracic aortic aneurysm. The patient was scheduled to undergo urgent surgery for a type A intramural hematoma and progressive aortic aneurysm; however, laboratory test results indicated blood clotting and cold agglutinin. Consequently, urgent surgery was rescheduled. After consulting with hematologists, rituximab was initiated 3 months before surgery, and plasmapheresis was performed 2 days before surgery for cold agglutinin disease. Under mild hypothermia conditions, total arch replacement using the frozen elephant trunk technique was performed while maintaining cerebral and lower body perfusion. The postoperative course was uneventful. On postoperative day 42, the patient was discharged without any neurological deficits.

**Conclusions:**

This case involving total arch replacement with mild hypothermia for an aortic arch aneurysm with cold agglutinin disease after rituximab treatment and plasmapheresis resulted in a successful outcome.

## Background

Cold agglutinin disease can lead to significant complications, especially for patients undergoing arch repair requiring hypothermic circulatory arrest. Rituximab and plasmapheresis are treatments for cold agglutinin disease. Therefore, after consulting with hematologists, we used rituximab and plasmapheresis before mild hypothermic aortic arch surgery to maintain the body temperature above the thermal altitude.

## Case presentation

**Fig. 1:**
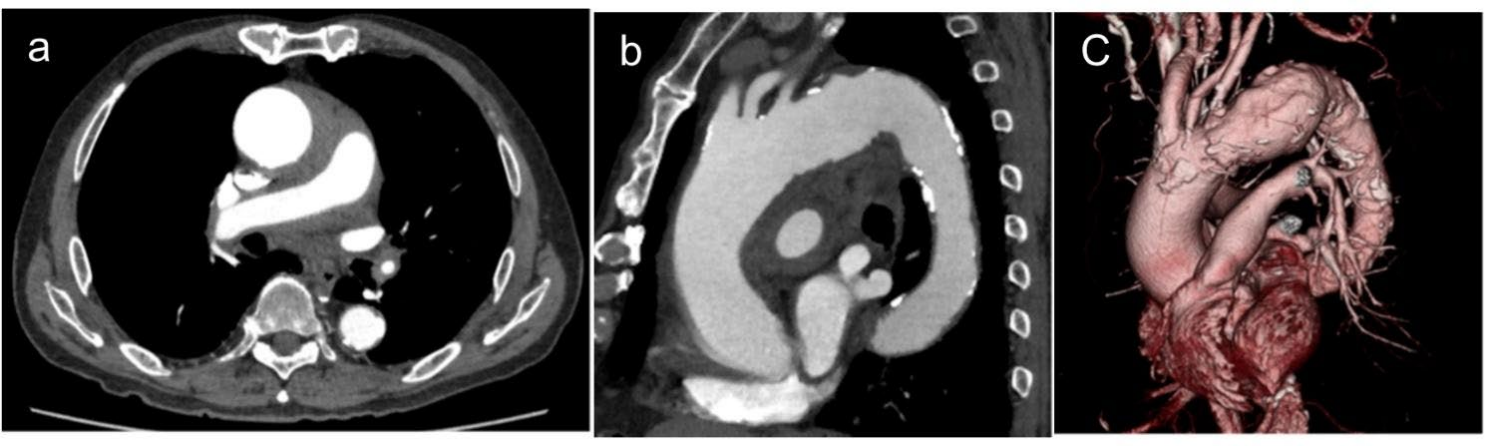
Acute type A aortic dissection with a non-communicating false lumen (a). The arch was dilated to 55 mm (b and c)

**Fig. 2:**
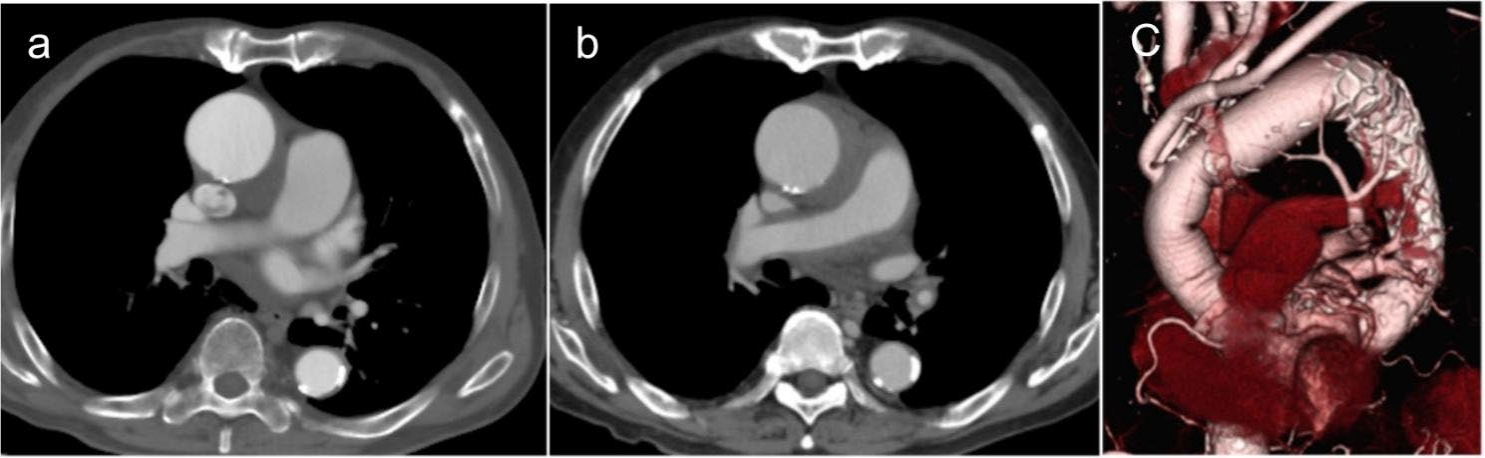
Acute type A aortic dissection with a non-communicating false lumen 3 months before surgery (a). Two day before surgery (b). Preoperative computed tomography image (c)

An 86-year-old man who reported chest pain was referred to our hospital and diagnosed with acute type A aortic dissection. He had a 13-mm intramural hematoma (IMH), an ascending aorta with a diameter of 50 mm, and shaggy descending aorta (Fig. 1a,b,c). His history included outpatient treatment for rheumatoid arthritis and a 55-mm thoracic aortic aneurysm. The patient was scheduled to undergo urgent surgery for the type A IMH and progressive aortic aneurysm; however, laboratory test results revealed blood clotting and cold agglutinin. His titers were 1:512 at 4 °C and 1:555 at 30 °C, and his thermal altitude was 32 °C. Surgery was rescheduled as an elective case because the IMH did not progress (Fig. 2a,b). We consulted hematologists regarding cold agglutinin disease (CAD) treatment. Weekly infusions of rituximab (375 mg/m^2^/week) were initiated 2 months before surgery for a total of 4 weeks, and plasmapheresis was performed 2 days before surgery. Cardiopulmonary bypass (CPB) was established through median sternotomy with cannulations on the ascending aorta because the thrombus in the ascending aorta was absorbed, and atherosclerotic lesions were observed from the descending aorta to the distal arch. Therefore, the ascending blood flow was selected because of the concern that cerebral infarction could be caused by the retrograde blood flow with femoral cannulation, and the prosthetic graft was anastomosed to the left axillary artery. Bicaval drainage was established, and warm blood antegrade cardioplegia was administered every 20 min.

**Fig. 3:**
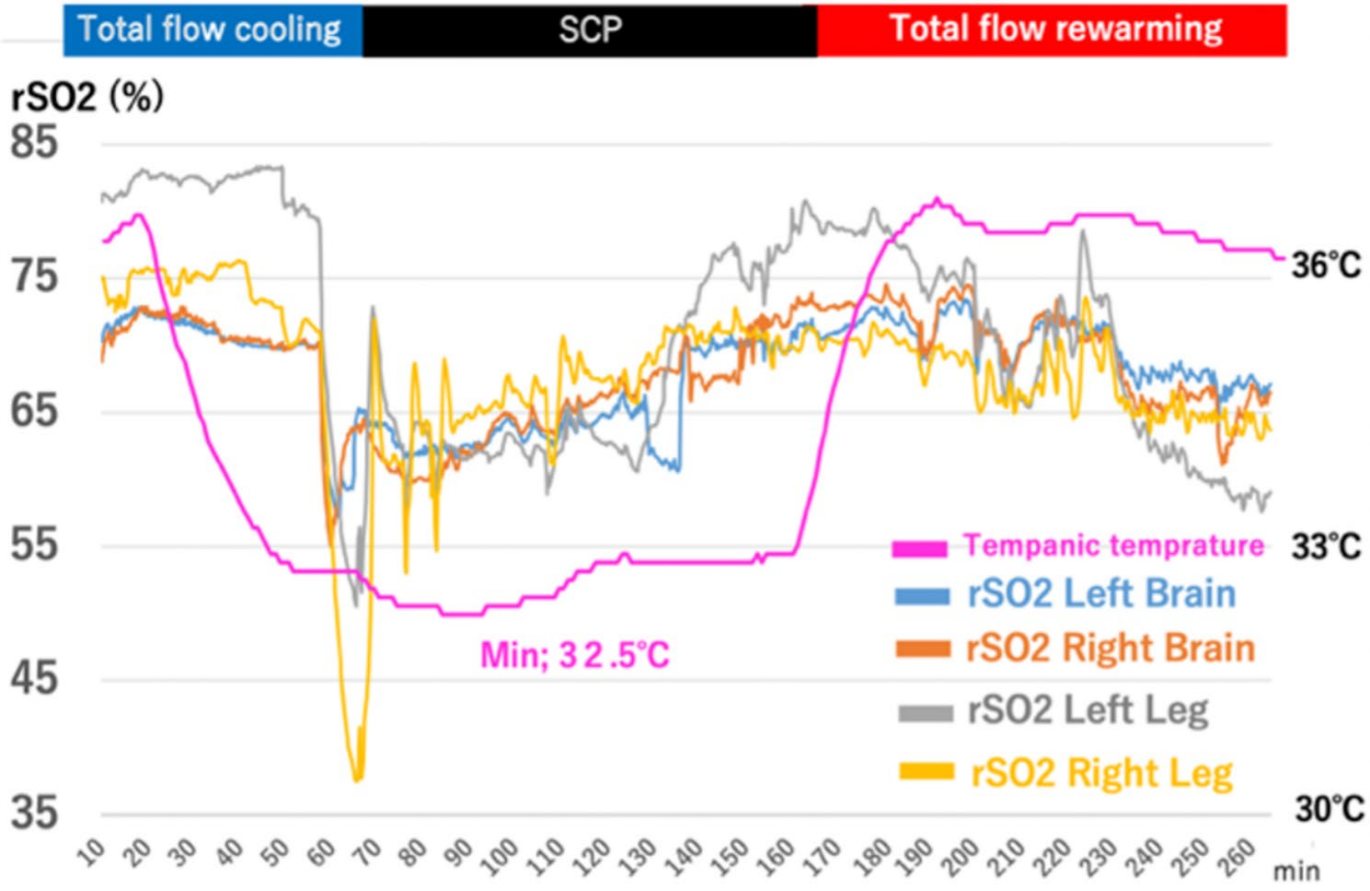
Regional oxygen saturation (rSO2) and tympanic temperature during surgery SCP, selective cerebral perfusion

Cardioplegic solution and blood transfusion should be performed while maintaining a temperature of 32 °C or higher. We discontinued CPB and initiated selective cerebral perfusion (SCP) from the left axillary artery at minimum tympanic and bladder temperatures of 32.5 and 33.2 °C, respectively. Subsequently, we opened the aneurysm and rapidly placed cannulas (SP-GRIPFLOW™; Fuji Systems, Tokyo, Japan) in two other carotid branches. Although 5 min was required to establish total SCP and right rSO_2_ decreased dramatically (Fig. 3), we avoided cerebral embolism during SCP by directly monitoring the two other cannulation sites and maintaining the SCP flow at 12 to 15 mL kg^-1^ min^-1^ and less than 60 mmHg.

A frozen elephant trunk (J Graft FROZENIX; Japan Lifeline, Tokyo, Japan) was deployed under the guidance of a transesophageal echocardiogram. After right femoral artery (FA) cannulation, a 12-Fr aortic occlusion balloon catheter (Reliant Stent Graft Balloon Catheter, Medtronic, Tokyo, Japan) was inserted to reduce the risk of spinal cord ischemia, and an arterial cannula was placed at the left FA for distal perfusion. The balloon was inflated to 25 mL in the frozen elephant trunk, but not in the shaggy descending aorta. The interval from the time we stopped CPB to the time we started retrograde perfusion via the FA (complete cardiac arrest time for the distal body) was 10 min. The flow rate was adjusted to approximately 0.7 to 1.5 L/min so that perfusion was performed at a mean pressure of 60 mmHg, as determined by near-infrared spectroscopy (NIRO-200NX; Hamamatsu Photonics KK, Hamamatsu, Japan). The normal physiological values of some cardiac patients are between 55% and 60% [1]. In this case, the value decreased by 40% at the time of circulatory arrest; however, it was recovered within a few minutes under lower body flow control (Fig. 3).

After completion of distal anastomosis with a 28-mm four-branch graft (Triplex; Terumo, Tokyo, Japan), anterograde perfusion of the lower body and rewarming were started through a graft branch. The duration of lower body perfusion from the left FA was 44 min. We transected the arch at zone 2 and reconstructed the left subclavian artery, left common carotid artery, and brachiocephalic artery, followed by proximal anastomosis (Fig. 2c). The total CPB and cardiac ischemic times were 230 min and 143 min, respectively. Postoperative bleeding occurred; therefore, 280 mL of fresh-frozen plasma and 480 mL of red blood cells were transfused in the intensive care unit. The peak serum lactate dehydrogenase and total bilirubin levels were 589 IU/L and 1.7 mg/dL, respectively, on postoperative day 10. Leukopenia was diagnosed on postoperative day 17, and improvement was confirmed. On postoperative day 42, the patient was discharged without any neurological deficits or infection.

## Discussion and conclusions

CAD is a form of autoimmune hemolytic anemia. Cold agglutinin is an autoantibody activated at temperatures below the normal physiological body temperature. Although these antibodies are present in the blood of most humans, they are rarely clinically important because they are not active at normal human body temperatures. CAD is characterized by the formation of cold agglutinin, which is fully activated at peripheral circulation temperatures. It is often caused by immunoglobulin M (IgM) autoantibodies activating antigens on the surface of erythrocytes under hypothermia conditions, thus causing hemolysis through aggregation and complement fixation [2]. CAD is classified as primary or secondary. The later conditions include genetic conditions [3], infections, autoimmune disorders, and malignancy. In this patient, rheumatoid arthritis can be the cause. On the other hand, there is a high possibility that it will be found by chance, and it may be important to deal with it. CAD is responsible for 16–32% of all autoimmune hemolytic anemia cases observed in children and adults, with an estimated prevalence of 10–16 cases per 1 million people [4–6]. Among these patients, 0.21% had cold antibodies that were identified before cardiovascular surgery or within 30 days after surgery [7]. The formation of cold agglutinin causes hemolysis and circuit obstruction, including obstruction of the cardioplegic fluid circuit; these are fatal complications [8]. In this case, blood agglutination occurred during the routine cross-match test that was performed in the transfusion department. At our hospital, the saline method for cross-matching is applied for IgM coagulation. If IgM is coagulated, then the presence of cold agglutin is possible; caution is required for these cases, as indicated by the transfusion department staff. As a result, we suspected CAD.

For cases such as ours, when there is time before surgery, it is advantageous to consult with hematologists to determine the optimal treatment for CAD. Rituximab administration by suppressing the production of IgM autoantibodies is recommended by the British Committee for Standards in Haematology for grade 1 C when the case is symptomatic [9]. After four courses, a response rate of 45–58% has been observed, with a complete remission rate of approximately 10% [10]. The conventional dose is 375 mg/m^2^ per week for 4 weeks.

Approximately 40% of patients experience mild-to-moderate infusion-related reactions, mostly fever and chills, during the first administration of rituximab [11]. Neutropenia has been observed in 2% of patients; occasionally, hypogammaglobulinemia is observed, but infections are not common [12].

**Fig. 4:**
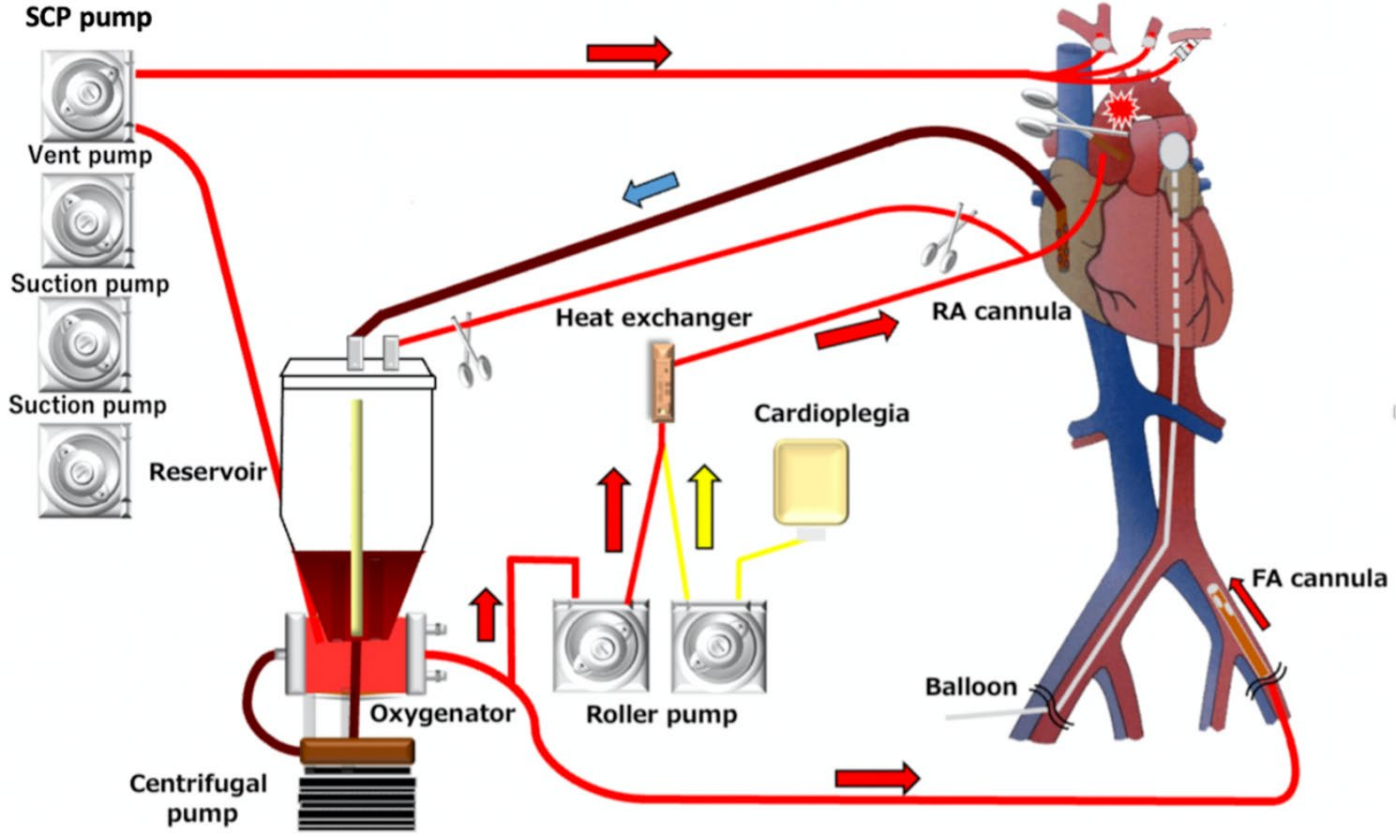
Perfusion technique for mild hypothermic total arch replacement for a thoracic aortic aneurysm with cold haemagglutinin disease. RA, right atrium; FA, femoral artery

Plasmapheresis is also an option for removing IgM autoantibodies [13], as recommended by the British Committee for Standards in Haematology for grade 2 C cases. IgM autoantibodies are present in blood vessels in approximately 95% of cases, and they have been reported to reduce agglutinin titers by up to 80% with plasmapheresis. Although it has been reported that the thermal amplitude is more important than the titer for predicting the likelihood of complications [14], the thermal altitude remained the same as that before the administration of rituximab and plasmapheresis; however, the titer decreased from 1:4 at 28 °C to 1:2 at 28 °C only after plasmapheresis (Fig. 4). Thermal management and cardioplegic strategies for CPB for cold agglutinin patients have been summarized by Barbara et al. [15].

Additionally, it has been reported that propofol has neuroprotective effects [16, 17]. Herein, the anesthesiologist selected propofol for this surgery; to avoid coagulation in the tube during CPB, we selected not only warm cardioplegia at which it was delivered was 32℃, but also recirculated warm cardioplegia solution (Fig. 4) for use in the circuit tube, except when cardioplegia was administered. The cardioplegia solution comprised 26 mEq potassium chloride added to 500 mL St. Thomas II solution.

Moreover, to avoid spinal cord ischemia, we performed total arch replacement with mild hypothermia and lower body perfusion with an occlusion balloon within the frozen elephant trunk [18].

Hemolysis progression after surgery has been reported [19]; therefore, we considered plasmapheresis. In this case, although the peak serum lactate dehydrogenase and total bilirubin levels had increased by postoperative day 10, they eventually normalized without any treatment.

Endovascular repair with debranching or revascularization of the aortic branches [20] may be considered an alternative for patients at high risk; however, thoracic endovascular aortic repair was not highly recommended for this patient because of his prior type A IMH and dilated ascending aorta (50 mm). Surgical arch replacement remains the gold-standard treatment for such complex conditions, and this method could serve as an alternative strategy when endovascular aortic repair is not feasible. As an algorithm for patients who require emergency surgery according to our experiment, the In cases where elective surgery is possible, such as false lumen closure and ascending aorta diameter of 50 mm or less without pericardial effusion, it is advisable to consult hematologists regarding the treatment of cold agglutinin disease.

This study had some limitations. First, it cannot be clearly understood whether rituximab and plasmapheresis were effective for CAD because it was not completely cured. Second, because CAD could be associated with rheumatoid arthritis, CAD remission did not occur. Therefore, other approaches, such as biologic agents for rheumatoid arthritis, may improve CAD; however, the response time may be longer. Third, sutimlimab, the first anti-complement antibody for cold agglutinin, was recently approved by the European Commission and United States Food and Drug Administration, and guidelines regarding the management of CAD can change. To our knowledge, this is the first reported case of total arch replacement with CAD after rituximab and plasmapheresis. Therefore, further research is needed.

In conclusion, a patient scheduled to undergo hypothermic thoracic aortic arch surgery was incidentally diagnosed with CAD. After consulting with hematologists, and after rituximab therapy and plasmapheresis were performed before surgery, arch replacement surgery under mild hypothermia conditions with CAD was safely performed.

## Data Availability

All data generated or analyzed during this study are included in this published article.

## List of abbreviations

CAD: Cold Agglutinin Disease
IMH: Intramural Hematoma
CPB: Cardiopulmonary Bypass
SCP: Selective Cerebral Perfusion
FA: Femoral Artery
